# Flow cytometric-based detection of CD80 is a useful diagnostic marker of acute myeloid leukemia in dogs

**DOI:** 10.3389/fvets.2024.1405297

**Published:** 2024-08-19

**Authors:** Tracy Stokol, Sophie Isabella Thomas, Martha Hoffman, Shay Zhao

**Affiliations:** Department of Population Medicine and Diagnostic Sciences, College of Veterinary Medicine, Cornell University, Ithaca, NY, United States

**Keywords:** acute leukemia, hematopoietic neoplasia, canine, immunophenotyping, flow cytometry, diagnostic testing, lymphoma, B7-1

## Abstract

**Introduction:**

CD80, a co-stimulatory molecule required for optimal T cell activation, is expressed on antigen-presenting cells, including monocytes and dendritic cells, in dogs and humans. We hypothesized that CD80 would be expressed on tumor cells in dogs from acute myeloid leukemia (AML) but not dogs with lymphoid neoplasms.

**Methods and results:**

We first evaluated the cellular staining pattern of a hamster anti-murine CD80 antibody (clone 16-10A1, ThermoFisher Scientific Cat# 17–0801-82, RRID: AB_469417) in blood and bone marrow aspirates from healthy dogs. Using flow cytometric analysis and examination of modified Wright’s-stained cytologic smears of unsorted and flow cytometric or immunomagnetic bead-sorted leukocytes, we show that the antibody binds to mature and immature neutrophils and monocytes, but not lymphocytes or eosinophils, in blood and bone marrow. We then added the antibody to routine flow cytometric panels for immunophenotyping hematopoietic neoplasms in dogs. We found that the antibody labeled tumor cells in 72% of 39 dogs with AML and 36% of 11 dogs with acute leukemia expressing lymphoid and myeloid markers (“mixed lineage”) but none of the dogs with B (*n* = 37) or T (*n* = 3*5*) lymphoid neoplasms. A higher proportion of tumor cells in dogs with AML were labeled with the anti-CD80 antibody vs antibodies against other myeloid-associated antigens, including CD4 (36%, *p* = 0.003), CD11b (44%), CD11c (46%), CD14 (38%, *p* = 0.006) and CD18 (59%, clone YFC118). In contrast, antibodies against CD11b and CD11c bound to tumor cells in 8–32% of the lymphoid neoplasms.

**Discussion:**

We show that CD80, as detected by antibody clone 16-10A1, is a sensitive and specific marker for AML and would be useful to include in flow cytometric immunophenotyping panels in dogs.

## 1 Introduction

Immunophenotyping with flow cytometry is a powerful tool used to help determine the cell lineage of hematopoietic neoplasms, including acute leukemia and lymphoma, in dogs. To identify normal and neoplastic canine leukocytes, diagnostic laboratories offer immunophenotyping panels, which typically include antibodies against the following surface antigens: T cell—CD3, CD5, CD4, CD8; B cell—CD21; monocyte—CD14; stem cell—CD34; common leukocyte—CD45; and major histocompatibility class II (MHCII), which is expressed on lymphocytes and monocytes in healthy dogs. Individual laboratories or investigators may also test for other antigens, such as CD22 (B cell) (1–3), CD25 (activated T and B cells or regulatory T cells) (4–8), CD11b and/or CD11c (neutrophils and monocytes) (2, 3, 9–12), CD61 (megakaryocytes) (3, 12, 13) and myeloperoxidase (an intracellular enzyme in neutrophils, eosinophils and monocytes) (9).

Acute leukemia in dogs is generally categorized as acute myeloid leukemia (AML), acute lymphoblastic leukemia (ALL), and acute undifferentiated leukemia (AUL). In humans, there are also acute leukemias of ambiguous lineage, which include AUL and mixed phenotype acute leukemia (MPAL), where the tumor expresses markers of more than one lineage on the same or different cell populations (14, 15). The entity of MPAL has not been definitively described in dogs, although we have seen dual expression of lymphoid and myeloid antigens in individual dogs with acute leukemia (2, 3). In dogs with acute leukemias, regardless of lineage, tumor cells often express the stem cell marker, CD34, while lacking MHCII (2, 3, 16), thus the different types are distinguished by expression of lineage-associated antigens. To diagnose an AML with flow cytometric analysis, tumor cells should express one or more of the myeloid-associated antigens (CD11b, CD11c, CD14, or CD4 without CD3 or CD5) (2, 3, 9–13, 16). Recently, a specific clone against human CD18 (YFC118.3) was found to be a useful antibody for the flow cytometric diagnosis of AML (16), because this clone primarily labels monocytes and neutrophils in normal dogs (17). A cut-off of >18% CD18^+^/MCHII^−^/CD4^−^ cells was used in a proposed algorithm to diagnose AML in dogs with an acute leukemia consisting of >10% or > 1 × 10^9^/L CD34^+^/MHCII^−^ cells (16). B- and T-ALL are distinguished by the solitary or combined expression of CD21 or CD22 and CD3 or CD5, respectively. However, it is difficult to distinguish B or T cell lymphoma from an ALL when tumor cells from lymphoma are found in high proportions in blood or bone marrow or both. Per WHO criteria, a blast cut-off of 25% in bone marrow is used to distinguish between a T and B precursor ALL from lymphoma (15). Acute leukemias that lack lineage-associated antigens are often categorized as AUL as a diagnosis of exclusion (11, 18). However, the term “acute leukemia-un-phenotyped” is preferred instead of AUL, because we lack antibodies against other lineage-associated markers in dogs (e.g., CD133, CD13, CD2, CD7, CD19), and cytochemical staining may help facilitate a diagnosis of AML in such cases (2, 3). It can be difficult to conclusively diagnose AML on flow cytometric analysis. Myeloid-associated antigens are not expressed on all cases of AML and may not be included in flow cytometric panels, which can result in a diagnosis of AML being based on lack of expression of classic lymphoid-associated antigens (usually CD3, CD5, and CD21) (13). There are only a limited number of antibodies that detect canine myeloid antigens and several of these antigens are also expressed on T cells, such as CD11d (19), or are intracellular antigens, requiring additional steps of fixation and permeabilization, such as myeloperoxidase (9). In addition, there is cross-lineage expression of markers, including CD5, CD22 and CD18, on leukemic cells in ALL and AML (2, 3, 16), which can confound determination of the involved lineage in an acute leukemia. Distinction between AML and ALL is important because dogs may be treated with different chemotherapeutic protocols, such as doxorubicin-cytosine arabinoside- and cyclophosphamide-doxorubicin-vincristine-prednisolone (CHOP)-based protocols, respectively (20, 21). To improve our ability to confidently diagnose AML on flow cytometric analysis, it would be useful to find antibodies against other myeloid antigens that could be added to standard flow cytometric panels.

CD80 (also known as B7.1/B7-1/BB1) is part of the B7 family of immunoglobulin-like proteins that is expressed on antigen-presenting cells, including monocytes, macrophages, and dendritic cells, in humans and mice. CD80 functions as a co-stimulatory molecule for T cells, via binding to CD28 or CD152 (CTLA4) in T cell membranes (22, 23). In dogs, CD80 expression has mostly been described in dendritic cells, including those from the skin, Peyer’s patches, and mesenteric lymph nodes, using flow cytometric analysis and immunostaining of tissue sections (24, 25). In addition, cytokine-stimulated cultured dendritic cells and polarized inflammatory macrophages derived from peripheral blood mononuclear cells (PBMCs) upregulate CD80 on flow cytometric analysis (26, 27). A subset of monocytes in blood express CD80 with flow cytometry (28). Since monocytic variants are the most common subtype of AML in dogs (2, 3, 11, 13), we reasoned that if CD80 is expressed on normal canine monocytes, it may be a helpful flow cytometric marker to confirm AML in a dog with acute leukemia. We performed a generic search for commercially available anti-CD80 antibodies that are cross-reactive for canine cells and found an Armenian Hamster anti-murine CD80 antibody (clone 16-10A1, ThermoFisher Scientific Cat# 17–0801-82, RRID: AB_469417). Preliminary testing showed this antibody labeled canine monocytes and, unexpectedly, neutrophils in residual blood samples that were submitted for routine hematologic analysis in the Clinical Pathology laboratory of the Animal Health Diagnostic Center (AHDC) at Cornell University. Even though the expression pattern was different than expected, given that the antibody bound to neutrophils and monocytes in canine blood, we hypothesized that when using this antibody, CD80 would be a flow cytometric marker of AML, but not lymphoid neoplasms, in the dog. We had these study objectives: (1) Verify the leukocyte labeling pattern of the anti-CD80 antibody using flow cytometric analysis on blood from healthy dogs, (2) Determine which cells in bone marrow aspirates from healthy dogs were labeled with the anti-CD80 antibody, and (3) Test our hypothesis by including the anti-CD80 antibody in flow cytometric panels used to immunophenotype canine hematopoietic neoplasms, including leukemia and lymphoma.

## 2 Materials and methods

### 2.1 Collection and source of samples

For objective 1, blood from healthy dogs was collected into EDTA anticoagulant-containing vacutainer tubes (BD Biosciences, Franklin Lakes, NJ, United States) by jugular or cephalic venipuncture. The healthy dogs were research animals housed at our institution or owned by students, faculty, and staff, and blood was obtained with owner consent. For objective 2, bone marrow was aspirated from the humeri of research beagles housed at our institution. Blood and bone marrow sample collection was approved by the Institutional Animal Care and Use Committee (#2009–0085). For objective 3, we used data from two sources: (1) Blood, aspirates from bone marrow, lymph node or masses, and body cavity fluid samples collected from dogs with hematopoietic neoplasia that were submitted to the AHDC for immunophenotyping as part of routine diagnostic service. These samples and data become the property of the AHDC after submission; (2) Blood, bone marrow or lymph node aspirates collected from dogs, with client consent, as part of an ongoing research study on acute leukemia. Data collection for the study herein ceased on July 6, 2023. Two immunophenotyping panels were used: One for acute leukemia and one for lymphoma. For routine immunophenotyping, the panel was chosen by the requesting clinician, with or without consultation with a clinical pathologist at our institution. Typically, lymphoma panels were requested for lymph node aspirates, acute leukemia panels were requested for bone marrow, mass or body cavity fluid aspirates, and both panels were run on blood samples. The acute leukemia panel was used for the research study. Each panel consisted of antibodies against various markers, with all panels including the anti-CD80 antibody. In cases phenotyped after April 2021, including all cases in the research study, we used triple-marker antibody combinations; before April 2021, the same antibodies were used as single markers with a few double-marker combinations (Tables 1, 2). Additional conjugated and unconjugated antibodies against other antigens were used as single markers in the acute leukemia panel, e.g., CD61-phycoerythrin (Beckman Coulter Cat# IM3605, RRID:AB 131237) and the α and β chain of the T cell receptor (TCRαβ, Peter Moore, clone CA15.8G7).

**Table 1:** Conjugated antibodies used for triple-labeling cells in acute leukemia immunophenotyping panels after April 2021, including their target antigen and registry number (when available) or source.

Target antigen	Registry number or source
CD3-FITC/CD4-PE/CD8-APC	Bio-Rad Cat# TC014, RRID:AB_808411
CD5-FITC/CD21-PE/CD45-APC	**CD5**: Bio-Rad Cat# MCA1037F, RRID:AB_322643; **CD21**: BD Biosciences Cat# 555422; RRID:AB_395816 **CD45**: Bio-Rad Cat# MCA1042APC, RRID:AB_324810
MHCII-FITC/CD34-PE/CD80-APC	**MHCII**: Bio-Rad Cat# MCA1044F, RRID:AB_322642; **CD34**: BD Biosciences Cat# 559369, RRID:AB_397238; **CD80**: ThermoFisher Scientific Cat# 17–0801-82, RRID: AB_469417.
CD4-FITC/CD14-PE/MHCII-A647	**CD4**: Bio-Rad Cat# MCA1038F, RRID:AB_321271, **CD14**: Agilent Cat# R086401, RRID:AB_579551; **MHCII**: Bio-Rad Cat#MCA1044A647, RRID: NA
CD11b-FITC*/CD22-PE or CD34-PE/CD11c-APC*	**CD11b**: Cell Signaling Technology Cat# 24442S, RRID: NA; **CD22:** Abcam Cat# ab23620, RRID:AB_447570, **CD11c:** BioLegend Cat# 117310 (also 117,309), RRID:AB_313779
CD11b-FITC/CD34-PE/CD18-A647*	**CD18**: Bio-Rad Cat# MCA503A647, RRID:AB_324799

This panel was used for routine diagnostic testing and for samples submitted as part of an acute leukemia research study. Before April 2021, the same antibody clones (exceptions noted) were used with the same or different fluorophores as single labels, with limited double labeling (MHCII and CD34 in some cases, CD14 and CD4, CD4 and CD8), for routine diagnostic testing. ^*^The antibodies against CD18 and CD11c were not used in all cases acquired after April 2021. In single color panels before April 2021, unconjugated antibodies were used to detect CD11b (Biorad Cat# MCA1777, RRID:AB_322922), CD11c (Bio-Rad Cat# MCA1778S, RRID:AB_3229420), and CD18 (Bio-Rad Cat# MCA1780, RRID:AB_2128639). Note the latter antibody is a different clone [CA1.4E9] from the anti-human CD18 antibody in the table and labels all leukocytes (29), so results for this antibody are not included in this study. A647, Alexa Fluor™ 647; APC, Allophycocyanin, FITC, Fluorescein isothiocyanate, NA, Not available, PE, Phycoerythrin.

**Table 2:** Conjugated antibodies used for triple-labeling cells in a lymphoma immunophenotyping panel used for routine diagnostic testing after April 2021, including their target antigen*.

Target antigen
CD3-FITC/CD4-PE/CD8-APC
CD5-FITC/CD21-PE/CD45-APC
MHCII-FITC/CD34-PE/CD80-APC
CD5-FITC/CD22-PE/CD25-A660

Before 2021, the same antibodies were used as single labels with the same or different fluorophores, with limited double labeling (MHCII and CD34 in some cases, CD4 and CD8). ^*^The same antibodies listed in Table 1 were used in this panel. Resource registry number CD25: Thermofisher Scientific Cat#50–0250-42, RRID:AB_10609350. A660, Alexa Fluor™ 660; APC, Allophycocyanin, FITC, Fluorescein isothiocyanate, PE, Phycoerythrin.

### 2.2 Antibody labeling of normal canine leukocytes in blood and bone marrow aspirates

Because the intended application of the antibody was for flow cytometric-based immunophenotyping, we used flow cytometric analysis to verify the labeling pattern of the anti-CD80 antibody in healthy canine blood in three ways: (1) Double- or triple-labeled analysis of normal dog leukocytes; (2) Flow cytometric-based sorting of anti-CD80 and -CD14 double-labeled leukocytes followed by cytologic analysis of sorted cells, using CD14 as a monocyte marker; and (3) Isolation of leukocytes followed by flow cytometric labeling with the anti-CD80 antibody. Monocytes, T cells and B cells were isolated by immunomagnetic bead-labeling, whereas neutrophils were isolated by double-density centrifugation followed by red blood cell (RBC) lysis. The same antibodies were used for verification of staining pattern of the anti-CD80 antibody and immunophenotyping of clinical cases (Table 1).

For the bone marrow aspirates, we labeled bone marrow mononuclear cells (BMMC) with the anti-CD80 antibody and then performed: (1) Flow cytometric analysis; and (2) Sorting for cells labeled with the anti-CD80 antibody followed by cytologic analysis of the positively and negatively stained sorted cells. All reagents were from Sigma-Aldrich (St Louis, MO, United States), unless otherwise specified.

#### 2.2.1 Double- or triple-labeled flow cytometric analysis of normal dog leukocytes with CD80 and monocyte, T and B cell markers

After lysis of RBCs with ammonium chloride, cells were resuspended in phosphate-buffered saline (PBS) containing 1% bovine serum albumin (BSA) and 0.05% sodium azide (PBSA). The following antibody combinations were then incubated with the cells for 30 min on ice with the anti-murine CD80-allophycocyanin (APC) antibody (0.01 mg/mL final antibody concentration): (1) Antihuman-CD14-phycoerythrin (PE) (final concentration 0.6 ug/mL) for monocytes; and (2) Anti- human CD21-PE (1:25 dilution) and anti-canine CD5-fluoroscein isothiocyanate (FITC) (1:100 dilution) for B and T cells, respectively. The cells were washed in PBSA and resuspended in PBS for analysis with a flow cytometer (BD FACSCalibur™, BD Biosciences), using appropriate compensation settings. Isotype and unlabeled cells were included in separate tubes. FloJo™ software was used for analysis (version 10, Ashland, OR, United States), excluding lysed RBCs and cellular debris. For the CD80 vs. CD14 double labels, the cells were separated into different gates (lymphocytes, monocytes and neutrophils), based on their characteristic forward (FSC) and side scatter (SSC) and the percentage of cells labeled with the anti-CD80 antibody (CD80^+^) and their CD80 median fluorescent intensity (MFI) was recorded for each gate. For the triple labels of CD80 vs. CD21 and CD5, all cells were examined for dual expression of CD80 and each lymphocyte antigen. The analysis was repeated on samples from 3 to 4 different dogs.

#### 2.2.2 Single-labeled flow cytometric analysis of canine bone marrow mononuclear cells

Bone marrow was aspirated into a syringe containing 1.5 mL 3.8% sodium citrate, filtered (70 μm, BD Biosciences), layered on a double-density gradient with 1.119 Histopaque and 1.077 Ficoll paque plus (Cytiva, Marlborough, MA, United States), and then centrifuged (400 x *g*, 30 min, 10°C), as we have previously described for harvesting PBMCs (30). The BMMC at the interface of the plasma and 1.077 density media was harvested, washed 4 times in PBS, and resuspended in PBSA. Then, 1 × 10^6^ cells were incubated with the anti-CD80 antibody for 30 min in PBSA, with unlabeled, isotype, and 7-aminoactinomycin D (7-AAD, for excluding dead cells) controls. The cells were washed in PBS for acquisition with an Accuri C6 BD Biosciences (the BD FACSCalibur^™^ was no longer available for use), then analyzed for labeling with the anti-CD80 antibody using a FSC vs. SSC plot on FloJo^™^ software.

#### 2.2.3 Flow cytometric-based sorting of cells labeled with the anti-CD80-antibody in blood and bone marrow

For blood and BMMC, 1 × 10^7^ cells were resuspended in PBS and incubated with the anti-CD80 and anti-CD14 antibodies (blood) or anti-CD80 antibody alone (BMMC) for 30 min on ice. The cells were resuspended in PBS with 1% BSA after a PBS wash and sorted into PBS with 10% BSA, using a MA900 (Sony Biotechnology, San Jose, CA, United States) or FACSAria^™^ III (BD Biosciences) flow cytometer (different sorters were used based on instrument availability) in the Cornell University BRC flow cytometry core facility (RRID:SCR_021740). Either 7-AAD or 4′,6-diamidino-2-phenylindole were used to exclude dead cells and single-labeled cells with isotype controls were used to define positive staining reactions during acquisition. For blood samples, double positive (CD80^+^/CD14^+^), single positive (CD80^+^ or CD14^+^), and double negative (CD80^−^/CD14^−^) cells were sorted. For BMMC, CD80^+^, CD80^−^ cells with high SSC (CD80^−^/high SSC), and CD80^−^ cells with low SSC (CD80^−^/low SSC) were sorted. Cytospin smears were prepared from the sorted cells after a 500 x *g* centrifugation step (with resuspension in PBS) and stained with modified Wright’s stain (Hematek 1,000, Siemens Healthcare Diagnostics Inc., Tarrytown, NJ, United States). A 100-cell differential cell count was done on each sorted population.

#### 2.2.4 Leukocyte isolation from blood followed by labeling with the anti-CD80 antibody

Peripheral blood mononuclear cells were harvested from the plasma/1.077 interface after double-density centrifugation of blood, as described for BMMC. B cells, T cells, and monocytes were then individually isolated from PBMC using conjugated antibodies against CD21, CD5, and CD14, followed by anti-murine IgG magnetic microbeads for CD14 and CD21 (Miltenyi Biotec Cat# 130–048-402, RRID:AB_244361) and anti-rat IgG magnetic microbeads for CD5 (Miltenyi Biotec Cat# 130–048-502, RRID:AB_244364) and a magnetic column (LS column, Miltenyi Biotec, Gaithersburg, MD, United States) as we described previously for isolating CD14^+^ monocytes (30). Isolated cells were then incubated with the anti-CD80 antibody, as described above for double- or triple-labeling, and flow cytometric analysis was performed to assess for dual expression of the markers. For neutrophil isolation, we harvested cells from the interface between the 1.077 and 1.119 gradients and lysed RBCs with 0.2% sodium chloride for 30 s, followed by quenching with 1.6% sodium chloride. This experiment was done on samples from 2 to 3 different dogs per cell type. We also performed differential cell counts on modified Wright’s-stained smears of cytospin preparations of the isolated cells.

### 2.3 Testing for CD80 expression in hematopoietic neoplasms

Flow cytometric panels, including the anti-CD80 antibody, were performed on blood and lymph node, bone marrow, mass or body cavity fluid aspirates from dogs with hematopoietic neoplasia, including lymphoma and leukemia, using BD FACSCalibur^™^, Accuri C6 (BD Biosciences) or Novocyte (2000R, Agilent Technologies, Santa Clara, CA, United States) flow cytometers. Various software was used to analyze the data, including BD FACScan software, FloJo^™^ and FCS Express (*De Novo* Software, Dotmatics, Pasadena, CA). In samples collected after July 2019, 7-AAD was used to gate out dead cells, otherwise cell debris was excluded from analysis based on its location in a FSC vs. SSC dot plot. A tumor cell gate was created in the FSC vs. SSC dot plots, based on abundance of events and characteristic location (low to high FSC, low to medium SSC). When possible, residual normal leukocytes (neutrophils, monocytes, lymphocytes) were gated separately in the same plot. Antibody labeling of each gate was assessed in histogram, SSC vs. fluorescence, and quadrant fluorescence plots for triple labels or histograms and quadrant plots for single or double labels. Only data from the region containing the tumor cells was included in this study; however, antigen expression on residual normal leukocytes was assessed as internal positive and negative controls for antibody staining. For all markers other than CD34, expression on ≥20% of gated tumor cells was considered positive, in relation to an isotype control (31, 32); for CD34, ≥5% labeling of the tumor cells was considered positive (2, 3, 33). Since monocytes and lymphocytes can overlap or fall in the tumor cell gate, to avoid including these cells in the analysis of CD80 expression, we compared the percentage of CD80^+^ cells with the percentages of CD14^+^ cells and monocytes in a differential cell count. We also assessed the location of CD80^+^ and CD14^+^ cells in FSC and SSC plots. When the percentage of CD80^+^ and CD14^+^ cells were similar (± 10%) and events overlapped in a typical location for monocytes in a FSC and SSC plot (medium to high FSC and medium SSC), they were considered residual monocytes. Similarly, when the tumor gate included residual normal lymphocytes (mostly in lymph node aspirates), we identified these normal cells based on their low FSC and SSC characteristics and flow cytometric results showing a mixed population of lymphocytes, i.e., CD21^+^/CD22^+^ B cells and CD3^+^/CD4^+^ and CD3^+^/CD8^+^ T cells.

Based on morphologic features and hematologic and flow cytometric results, the hematopoietic neoplasms in the dogs were classified as B or T lymphoma/leukemia, B or T chronic lymphocytic leukemia (CLL), acute myeloid leukemia (AML), or “mixed lineage” leukemia. Classification of lymphoid neoplasms as B or T was based on tumor cell expression of CD21^+^ or CD22^+^ or both and CD3^+^ or CD5^+^ or both, respectively. In select cases, immunocytochemical staining for CD3 was performed on blood or cytology smears to confirm a T cell origin or verify weak or negative flow cytometric reactions for CD3, with ≥20% CD3^+^ tumor cells being defined as a positive reaction. B or T cell lymphoma/leukemias consisted of intermediate to large cells (“blasts”) and were grouped as a single entity, regardless of the blast percentage in blood or bone marrow. Chronic lymphocytic leukemia was characterized by a mature lymphocytosis, with negative test results for tick-borne diseases. For B-CLL, we used previously defined criteria of > 5.0 × 10^9^ small lymphocytes/L with > 60% CD21^+^ cells (34). Similar criteria are lacking for T-CLL in dogs, so we used a cut-off of > 20.0 × 10^9^ lymphocytes/uL with > 60% CD3 or CD5 expression. An acute leukemia was classified as AML on flow cytometric analysis if there were ≥ 20% blasts in blood or bone marrow, lymph node, tissue, or body cavity fluid aspirates and blasts were positive for myeloid-associated antigens CD4, CD11b, CD11c, CD14, and CD18, alone or in combination and negative for B and T cell markers (3). Because myeloid cells (monocytes, neutrophils, and eosinophils) typically comprise <5% of cells in normal lymph node aspirates (33, 35), we defined an extramedullary AML in lymph node aspirates as ≥20% blasts in cytologic smears combined with ≥5% positive reactions for myeloid-associated antigens on flow cytometric analysis. In cases in which we performed triple labeling with CD34/CD80/MHCII, CD34/CD11b/CD11c or CD34/CD11b/CD18, a leukemia was also classified as AML if there were ≥ 5% CD34^+^/CD11b^+^, CD34^+^/CD11c^+^, or CD34^+^/CD18^+^ cells. In acute leukemias that could not be phenotyped by flow cytometry, cytochemical staining was done to distinguish between AML and ALL, as described (2, 3, 36). Expression of alkaline phosphatase (ALP), alpha-naphthyl butyrate esterase (ANBE), chloroacetate esterase (CAE), myeloperoxidase (MPx), and Sudan Black B (SBB) in ≥3% of the cells, alone or in combination, was consistent with a myeloid lineage for an acute leukemia (Supplementary Table S1) (2, 3). AML was further classified into “not otherwise-specified” categories of unclassified, myelomonocytic, monocytic/monoblastic, and megakaryoblastic leukemia (Supplementary Table S2) (14, 15). A “mixed lineage” leukemia was diagnosed if blasts were positive for myeloid- and lymphoid-associated antigens on flow cytometric analysis or expressed lymphoid antigens on flow cytometric analysis but had cytochemical staining reactions typical of AML.

### 2.4 Statistical analysis

Normality was assessed with a Shapiro–Wilk test when there was more than 3 data points per group. Non-Gaussian data was described as median and range, whereas 3 data points per group were described as mean and range. Medians of two groups were compared with a Wilcoxon signed-rank test (e.g., median percentage of anti-CD80-labeled monocytes vs. neutrophils). Proportions were compared with a Fisher’s exact test with a Bonferroni correction for the number of pairwise comparisons. Significance was set at *p* < 0.05.

## 3 Results

### 3.1 Binding of the anti-CD80 antibody to peripheral blood leukocytes from normal dogs

**Figure 1:**
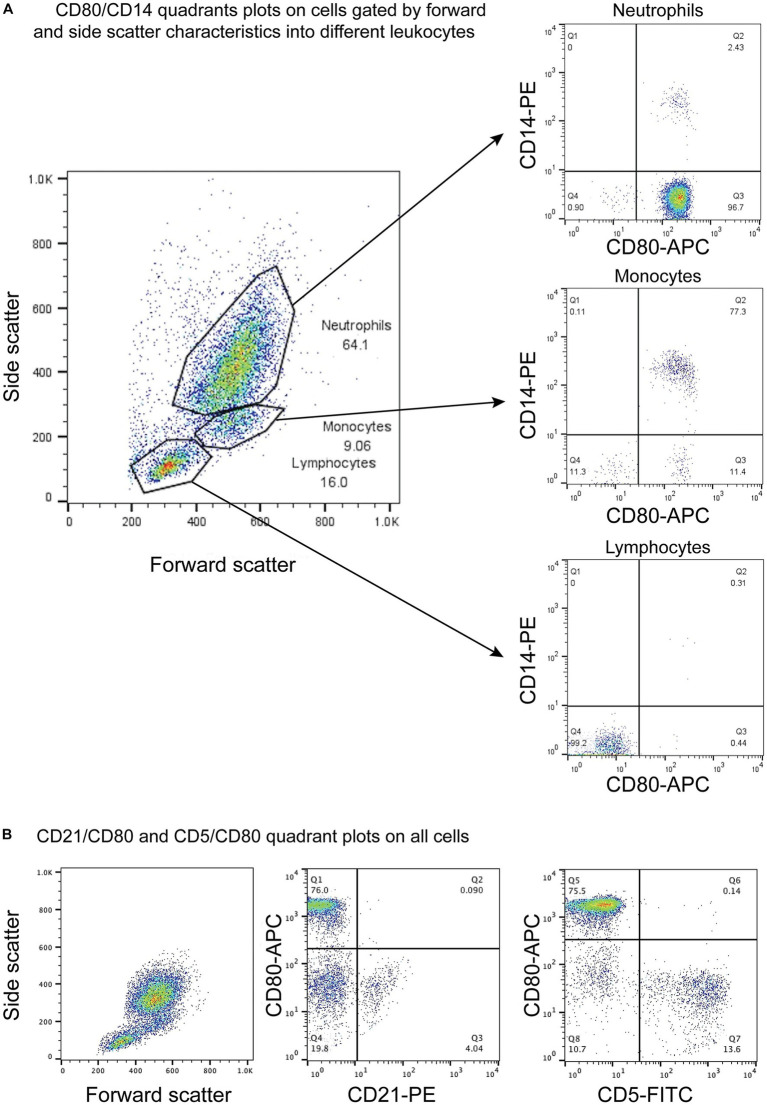
Flow cytometric dot plots of anti-CD80 antibody labeling of leukocytes in blood from healthy dogs. **(A)** Three different cell populations were identified on a forward and side scatter plot, corresponding to neutrophils, monocytes, and lymphocytes. The cells were double-labeled with anti-CD80-APC and -CD14-PE antibodies, with CD14 being used as a monocyte marker. CD80 was only expressed on CD14^+^ monocytes and CD14^−^ neutrophils but not lymphocytes (CD80^−^/CD14^−^). The few CD14^+^ and CD14^−^ cells in the neutrophil and monocyte gates likely represent low numbers of monocytes and neutrophils in the respective gates. The CD80^−^/CD14^−^ cells in neutrophil and monocyte gates could be large lymphocytes (representative result from 1 of 4 dogs). **(B)** Triple-labeling of dog leukocytes with CD80-APC, CD21-PE and CD5-FITC shows that CD21^+^ B cells and CD5^+^ T cells are CD80^−^ (representative result from 1 of 3 dogs). All leukocyte events were combined for analysis vs. splitting the events into different gates based on forward and side scatter.

CD80, as detected with the clone in this study, was consistently expressed on >95% of cells gated as monocytes (CD14^+^) and neutrophils (CD14^−^) in blood samples taken from 4 different healthy dogs. The intensity of CD80 expression (MFI) in CD14^+^ monocytes and CD14^−^ neutrophils was similar (Table 3). In contrast, cells gated as lymphocytes in the CD80 vs. CD14 experiments or triple-labeled with CD80 and B and T cell markers, CD21 and CD5, respectively, were negative for CD80 (Figure 1, representative images from one dog for CD80 vs. CD14 and a different dog for CD80 vs. CD21 or CD5). Events gated as lymphocytes in the CD80 vs. CD14 experiments had a median CD80 MFI of 6 units (range, 1–9 units), which was not significantly different from the median CD80 MFI of 5 units (range, 4–7 units) for the isotype control in that gate (*p* = 0.750).

**Table 3:** Labeling of leukocytes in the blood of healthy dogs with the anti-CD80 antibody, expressed as a percentage and median fluorescent intensity (MFI) of CD14^+^ monocytes and CD14^−^ neutrophils in regions gated as monocytes and neutrophils, respectively, in a forward and side scatter dot plot (see Figure 1) (*n* = 4).

	CD80^+^ percentage*		CD80^+^ MFI (units)*	
Gated cells	Median	Range	Median	Range
CD14^+^ monocytes	100	97.9–100	153	93–204
CD14^−^ neutrophils	98.6	96.3–99.9	132	99–208

Cells gated as lymphocytes did not label with the anti-CD80 antibody; their MFI (median, 6 units) was similar to the isotype control (median, 5 units) (*p* = 0.750). ^*^The median percentage of CD80^+^ cells and CD80 MFI in CD14^+^ monocytes and CD14^−^ neutrophils was not significantly different (*p* > 0.05).

**Figure 2:**
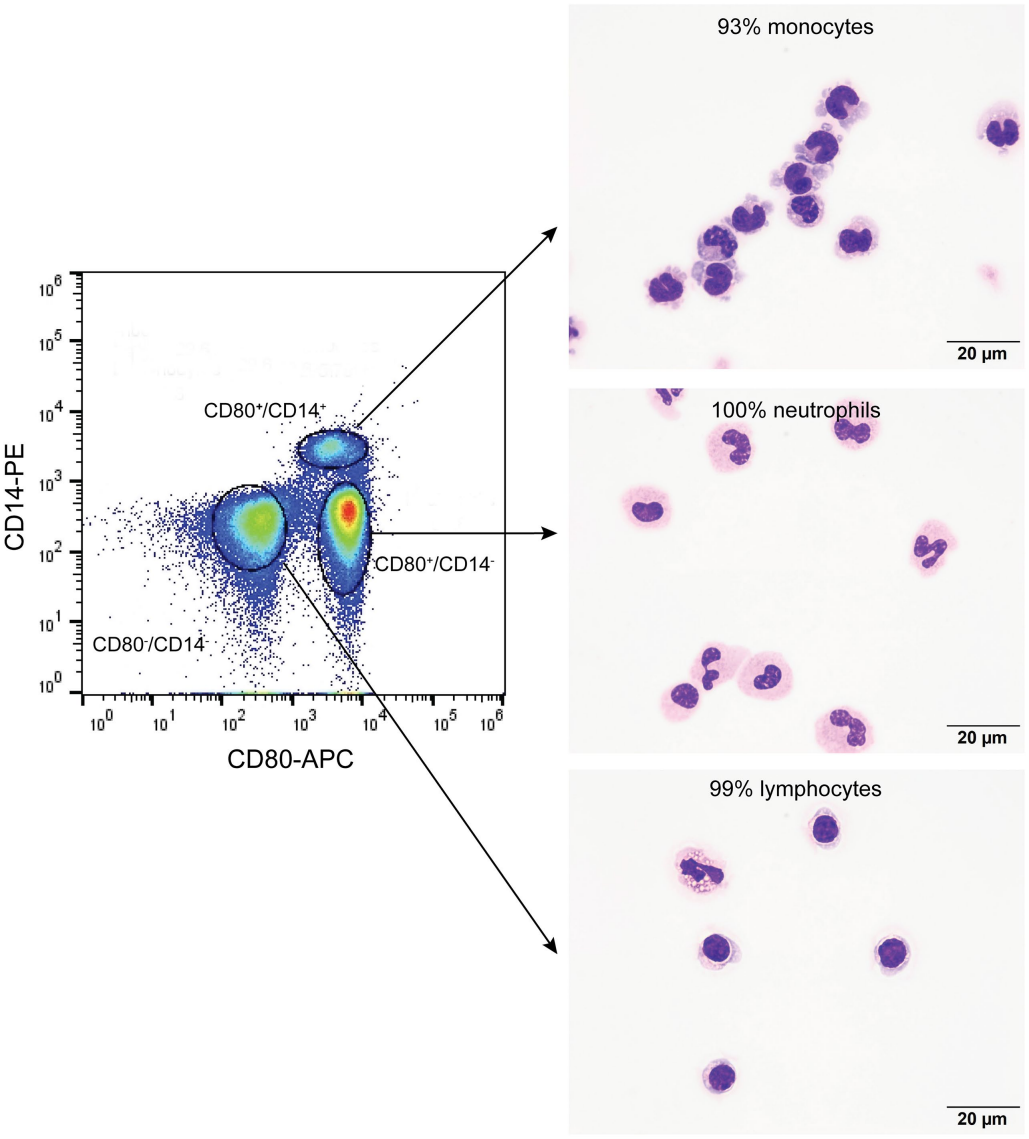
Flow cytometric sorting of peripheral blood leukocytes double-labeled with the anti-CD80 antibody and an anti-CD14 antibody into CD80^+^/CD14^+^, CD80^+^/CD14^−^, and CD80^−^/CD14^−^ populations (left panel) with corresponding images of modified Wright’s-stained smears and percentages of the predominant cell from differential cell counts of cytospin smears of the sorted populations (right panel, scale bar = 20 μm). CD80^−^/CD14^+^ cells were not identified or sorted. The black circles indicate sorted events (a tight gate was chosen to minimize contamination from other populations; representative image from 1 of 3 dogs). CD80^+^/CD14^+^ sorted cells were mostly monocytes, a few of which contained low numbers of cytoplasmic vacuoles. The CD80^+^/CD14^−^ sorted cells were mostly neutrophils, many of which lacked nuclear segmentation or were undergoing pyknosis, which we attributed to the sorting procedure. The CD80^−^/CD14^−^ sorted cells were mostly lymphocytes, with a few eosinophils.

On flow cytometric sorting with CD80- and CD14-labeled cells, only three populations were evident: CD80^+^/CD14^+^, CD80^+^/CD14^−^, and CD80^−^/CD14^−^ cells. No cells were CD80^−^/CD14^+^, suggesting that all CD14^+^ monocytes in blood from healthy dogs are CD80^+^. Differential cell counts on modified Wright’s-stained smears showed that monocytes, neutrophils, and lymphocytes dominated in the CD80^+^/CD14^+^, CD80^+^/CD14^−^ and CD80^−^/CD14^−^ populations, respectively (Figure 2, Table 4). Eosinophils were identified in the CD80^−^/CD14^−^ cells, indicating they are negative for CD80, when using the anti-hamster antibody (Table 4). Since eosinophils are found in low concentrations in blood and cannot be distinguished from neutrophils based on scatter characteristics or antibodies used in flow cytometric panels, their lack of binding of the anti-CD80 antibody was only uncovered by cell sorting.

**Table 4:** Percentage differential cell counts (mean and range) from modified Wright’s-stained cytospin smears of three cell populations sorted by flow cytometry using anti-CD80 and -CD14 antibodies in the blood of healthy dogs (*n* = 3): CD80^+^/CD14^+^, CD80^+^/CD14^−^ and CD80^−^/CD14^−^.

Sorted cells			
Leukocyte	CD80^+^/CD14^+^	CD80^+^/CD14^−^	CD80^−^/CD14^−^
Neutrophil %	2 (1–4)	97 (93–100)	1 (0–1)
Lymphocyte %	2 (0–5)	2 (0–5)	80 (63–99)
Monocyte %	96 (93–97)	1 (0–1)	1 (0–2)
Eosinophil %	0 (0–0)	0 (0–0)	18 (1–35)
Basophil %	0 (0–0)	0 (0–1)	0 (0–0)

A CD80^−^/CD14^+^ population was not identified (see Figure 2).

**Figure 3:**
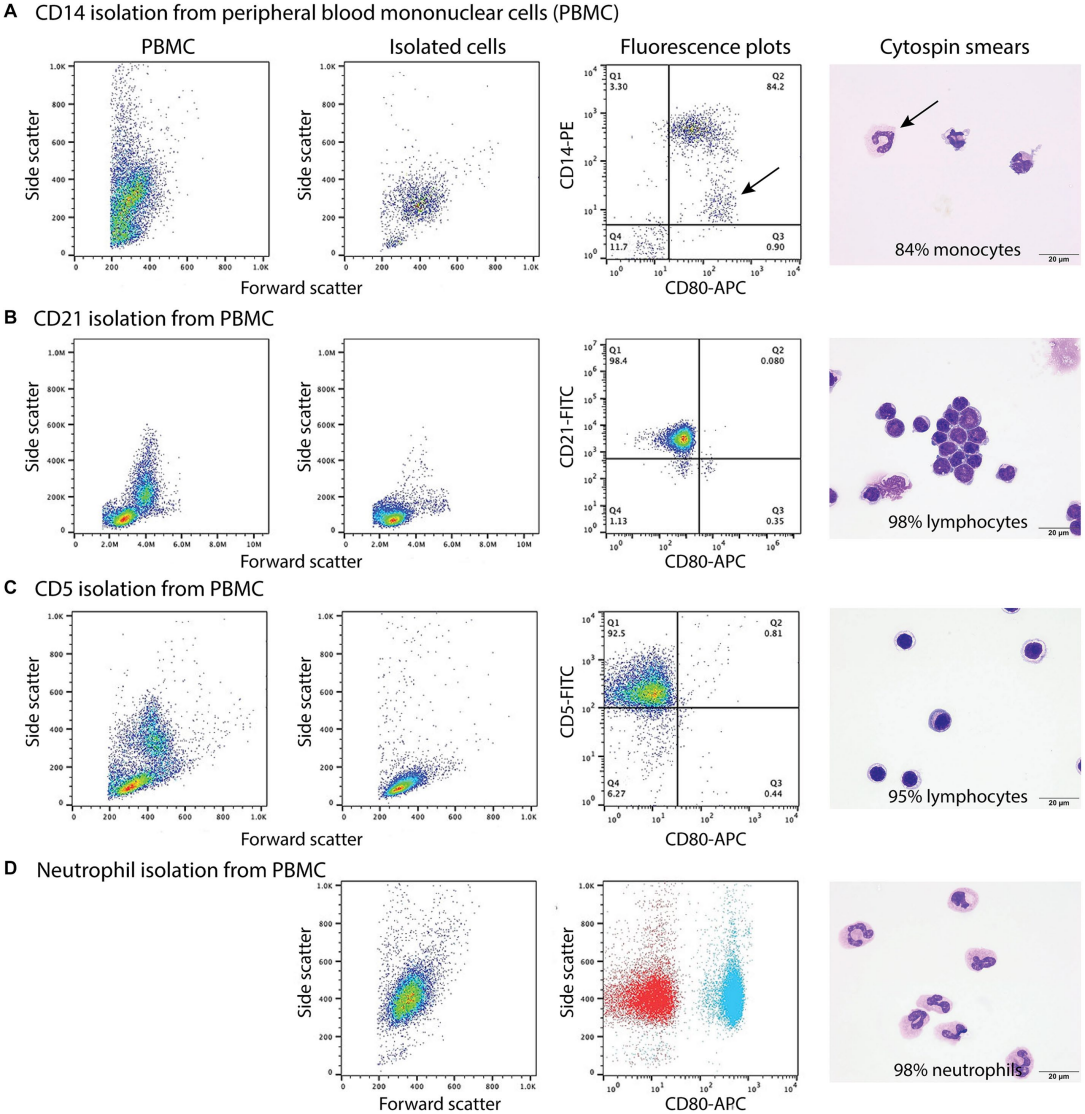
Labeling of monocytes, B cells, T cells, and neutrophils isolated from the blood of healthy dogs (representative results from 1 of 2–3 dogs for each cell type) with the anti-CD80 antibody. **(A-C)** Monocytes, B cells, and T cells were isolated from peripheral blood mononuclear cells (PBMCs) using immunomagnetic beads and anti-CD14-PE, -CD21-FITC, and -CD5-FITC antibodies, respectively. The first panel shows the forward and side scatter events in PBMCs while the second panel shows the forward and side scatter events of isolated cells. The third panel is a fluorescent quadrant plot of double-labeled cells after adding the anti-CD80-APC antibody. The fourth panel shows a representative modified Wright’s-stained image of a cytospin smear of the isolated cells on which 100-cell differential cell counts were done (scale bar = 20 μm). **(A)** CD14-PE-isolated cells were mostly CD80^+^ monocytes (84% of a differential cell count), with a few contaminating neutrophils (10%) that were weakly CD14^+^ (arrows, third and fourth panels). A few monocytes had cytoplasmic vacuoles (fourth panel). Lymphocytes were negative for CD80^−^/CD14^−^ (lower left quadrant, third panel, 6%). **(B)** CD21-FITC-isolated cells were mostly lymphocytes, which were CD80^−^ (third panel). Lymphocytes were primarily small cells, some of which had clefted or convoluted nuclei (variants of normal), with a few small or large reactive forms (fourth panel). **(C)** CD5-FITC-isolated cells were mostly lymphocytes, which were CD80^−^ (third panel). Lymphocytes were small cells with a few large or reactive forms. Several lymphocytes had a few clear cytoplasmic vacuoles, which could be due to the isolation procedure (fourth panel). **(D)** Neutrophils were isolated from the 1.077/1.119 interface of the double-density gradient used to obtain PBMCs and were single-labeled with the anti-CD80-APC antibody. The first panel shows a forward vs. side scatter plot of the isolated neutrophils and the second panel is a CD80 fluorescence vs. side scatter dot plot (blue) with overlaid hamster-APC isotype (red), showing neutrophils are CD80^+^. The third panel shows a representative modified Wright’s-stained image of a cytospin smear of the isolated cells, which were primarily segmented neutrophils. The vacuolated cytoplasm in one neutrophil is likely an artifact of the isolation procedure (scale bar = 20 μm).

When monocytes, B cells, and T cells were isolated via magnetic bead labeling with the anti-CD14, -CD21 and -CD5 antibodies then labeled with the anti-CD80 antibody, only CD14^+^ monocytes were CD80^+^, corroborating the results of the multiple labeling and cell sorter experiments. The purity of the isolated cells was generally >80% (Figures 3A–C, Table 5). Contaminating neutrophils in the CD14-magnetic bead isolated cells were weakly CD14^+^, suggesting that neutrophils were activated by the procedure and either expressed CD14 or had bound CD14^+^-microparticles shed from activated monocytes (Figure 3A). Similar to monocytes, neutrophils isolated from the double-density gradient were CD80^+^ (Figure 3D) with a purity of 78% or higher (Table 5).

**Table 5:** Percentage differential cell counts (mean and range) from modified Wright’s-stained cytospin smears of isolated monocytes, B cells, T cells, and neutrophils.

Leukocyte	Isolated cells			
	CD14^+^ monocytes	CD21^+^ B cells	CD5^+^ T cells	Neutrophils
Neutrophil %	5 (1–10)	11 (0–33)	5 (1–11)	88 (78–98)
Lymphocyte %	5 (4–6)	80 (42–100)	93 (88–97)	9 (0–16)
Monocyte %	90 (84–95)	4 (0–12)	1 (0–2)	0 (0–1)
Eosinophil %	0 (0–0)	5 (0–13)	1 (0–2)	2 (1–4)
Basophil %	0 (0–0)	0 (0–0)	0 (0–0)	0 (0–1)

Monocytes, B cells, and T cells were isolated by magnetic bead labeling using antibodies against CD14, CD21, and CD5, respectively (*n* = 3 for CD14 and CD5 and *n* = 2 for CD21). Neutrophils were isolated from the lower interface of the double-density gradient (*n* = 3).

### 3.2 Binding of the anti-CD80 antibody to bone marrow mononuclear cells from normal dogs

**Figure 4:**
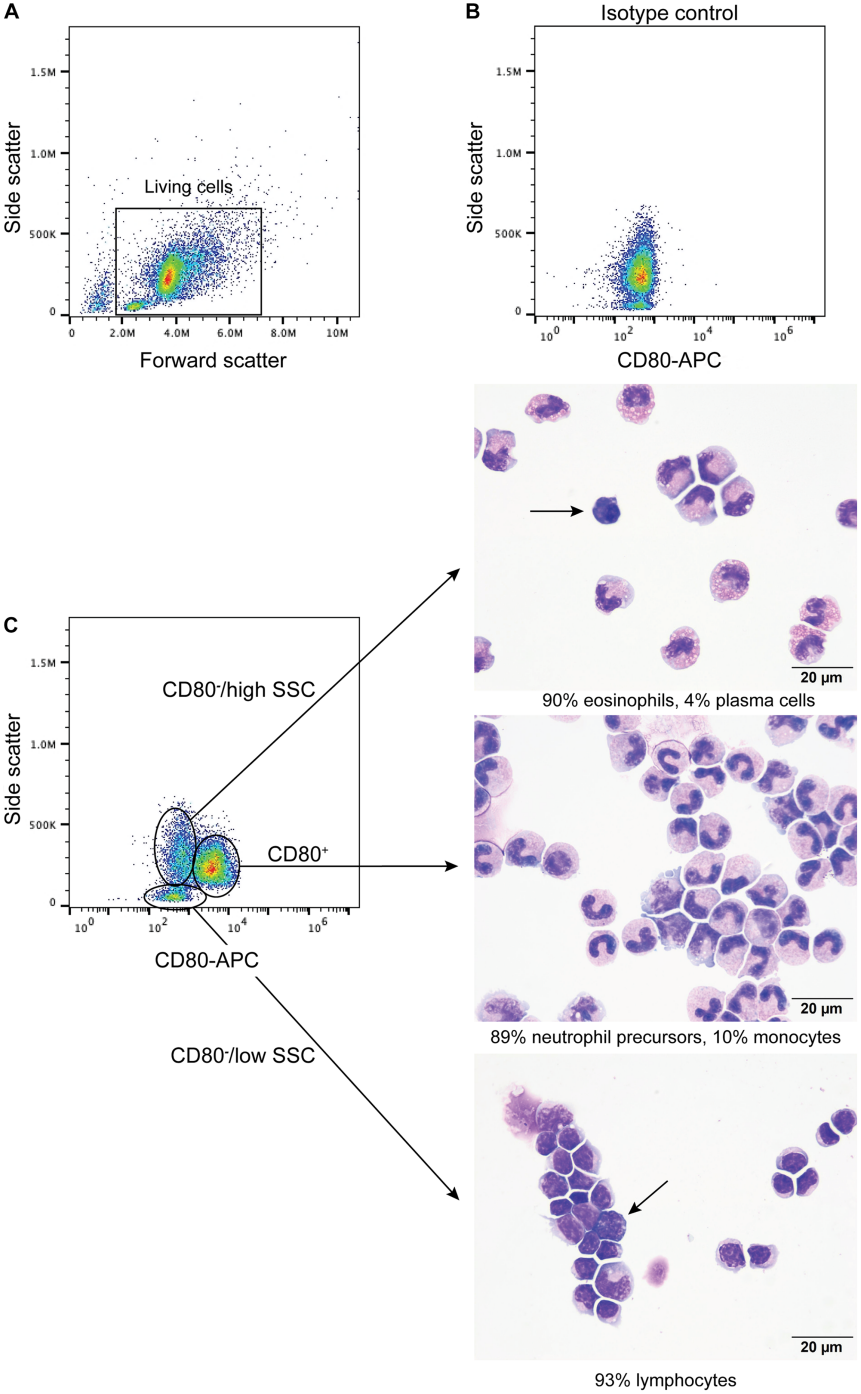
Labeling of bone marrow mononuclear cells from healthy dogs with the anti-CD80 antibody. Mononuclear cells were isolated from the plasma/1.077 interface of a double-density gradient after centrifugation of bone marrow aspirates from healthy dogs and labeled with the anti-CD80 antibody. After excluding dead cells (based on 7-AAD expression), lysed red blood cells and debris **(A)**, the living cells were evaluated for anti-CD80 antibody binding, using an isotype control **(B)** to determine positive labeling. A single population of CD80^+^ cells with high side scatter (SSC) was identified in a CD80 fluorescence vs. SSC plot, with two CD80^−^ populations, of high and low SSC (**C**, representative results from 4 experiments from 2 different dogs). The three different populations were then sorted from BMMC of one dog and differential cell counts were performed on modified Wright’s-stained cytospin smears of the sorted cells **(C)**. The CD80^+^ cells were mostly neutrophil precursors and monocytes (differential cell count: 83% band neutrophils, 4% metamyelocytes, 2% myelocytes, 10% monocytes, and 1% lymphocytes). The CD80^−^/high SSC fraction were mostly mature and immature eosinophils. Plasma cells were only seen in this fraction (arrow) (differential cell count: 90% eosinophils, including bands, metamyelocytes and myelocytes, 2% lymphocytes, 4% monocytes, and 4% plasma cells). The CD80^−^/low SSC fraction were mostly lymphocytes with fewer monocytes (differential cell count: 93% lymphocytes and 7% monocytes). Lymphocytes included small and large reactive forms, with deep blue cytoplasm and convoluted nuclei (arrow).

When BMMC were incubated with the anti-CD80 antibody, a single CD80^+^ population with high SSC was identified (Figure 4) and comprised a median of 66% (range, 61–71%) of the living cells (*n* = 4). Typically, neutrophils and monocytes have higher SSC than lymphocytes. There were two populations of CD80^−^ cells; (1) One had similar high SSC to the CD80^+^ cells and comprised a median of 16% (range 16–23%) of living cells, and (2) Another population with low side SSC, corresponding to cells with less complexity, such as lymphocytes (Figure 4), comprising a median of 13% (range 11–21%) of living cells. When differential cell counts were done on modified Wright’s-stained cytospin smears of these 3 flow cytometric-sorted populations, the CD80^+^ cells consisted of neutrophil precursors (bands to myelocytes, with rare progranulocytes) and monocytes (Figure 4C). In contrast, the CD80^−^/high SSC cells were mostly mature and immature eosinophils with fewer monocytes, large reactive lymphocytes, and plasma cells (Figure 4C). Rare progranulocytes and basophils were seen. Lymphocytes comprised the majority of the CD80^−^/low SSC cells, with fewer monocytes, and there were reactive lymphocytes in this population (Figure 4C). Segmented neutrophils were not seen in any fraction because these cells settle at the interface between the 1.077 and 1.119 density gradients. The BMMC sorting showed that the anti-CD80 antibody binds to mature and immature neutrophils and affirms that it does not bind to mature and immature eosinophils. In addition, the antibody may not detect plasma cells. It is difficult to determine whether the antibody labels progranulocytes and basophils, given that these cells were only seen in low numbers in the cytospin smears and progranulocytes were identified in both CD80^+^ and CD80^−^ high scatter fractions.

### 3.3 Binding of the anti-CD80 antibody to tumor cells in dogs with hematopoietic neoplasms

**Figure 5:**
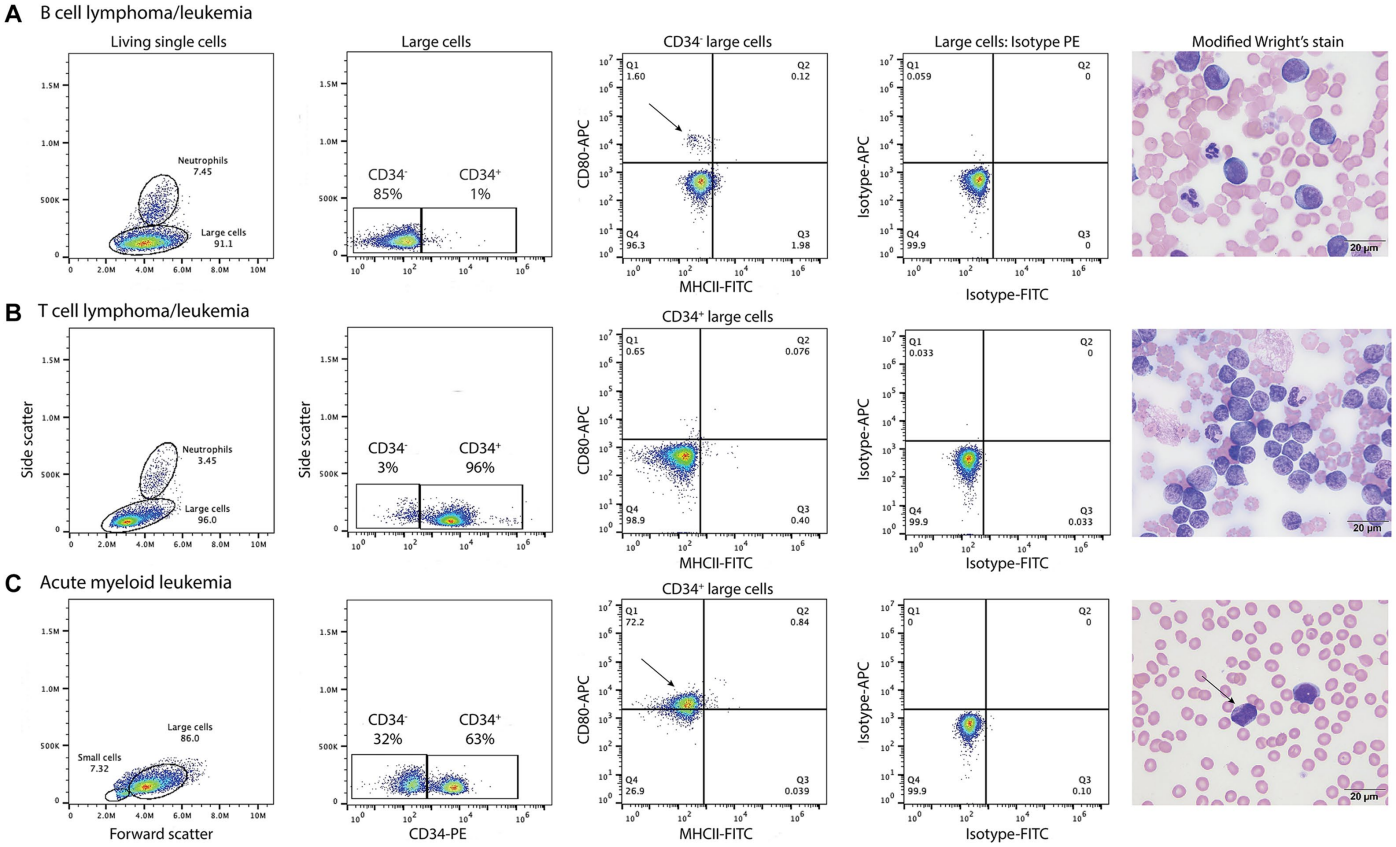
Labeling of tumor cells with the anti-CD80 antibody in dogs with hematopoietic neoplasia. Representative flow cytometric (first four panels) and modified Wright’s-stained images (fifth panel, scale bar = 20 μm) of venous blood in one case each of B cell lymphoma/leukemia **(A)**, T cell lymphoma/leukemia **(B)**, and acute myeloid leukemia (AML, **C**). Gated tumor events were intermediate to large (large cells) in forward vs. side scatter plots (first panel) and were assessed for positive labeling with anti-CD34-phycoerythrin (PE), CD80-allophycocyanin (APC), and major histocompatibility II-fluorescein isothiocyanate (MHCII-FITC) antibodies. The large cell gate was separated into CD34^+^ and CD34^−^ events using a CD34-PE vs. side scatter plot (second panel). Quadrant plots of MHCII-FITC vs. CD80-APC were then used to further define the CD34^−^ **(A)** and CD34^+^ **(B,C)** cells (third panel). Isotype controls were used to set the quadrant regions (fourth panel). **(A)** The tumor cells in venous blood (large cell gate) from a dog with B cell lymphoma/leukemia were negative for CD34^−^, CD80^−^ and MHCII^−^. The dog had 87% blasts in blood, which were large cells (11–14 μm) with round nuclei containing lightly stippled chromatin and up to 5 prominent nucleoli. The cells had a small amount of deep blue cytoplasm with a perinuclear clear zone. A small population of CD80^+^/MHCII^−^ cells (arrow, third panel) likely represent neutrophils inadvertently included in the large cell gate. **(B)** The tumor cells in venous blood from a dog with T cell lymphoma/leukemia were CD34^+^ but CD80^−^/MHCII^−^. The tumor cells comprised 89% of the cells in blood and were intermediate to large (9–14 μm) with round to deeply convoluted nuclei containing lightly clumped chromatin and 1–2 indistinct nucleoli. They had a scant to small amount of medium blue cytoplasm. **(C)** In venous blood from a dog with AML, 63% of the tumor cells (large cells) were CD34^+^. Of the CD34^+^ cells, 72% were CD80^+^/MHCII^−^ (arrow, third panel). The dog had 79% blasts in blood, which were mostly intermediate to large cells (10–14 μm) with round to oval nuclei containing lightly stippled chromatin and 1–2 nucleoli. They had a small amount of light to medium blue cytoplasm and 5% of blasts contained purple or red cytoplasmic granules (arrow, fourth panel). See Supplementary Figure S1 for results from the neutrophil gated region in the dog with B lymphoma/leukemia and the small cell region in the dog with AML.

B cell neoplasms were identified in 37 dogs from blood (*n* = 7) or lymph node (*n* = 27), bone marrow (*n* = 2), or pleural fluid (*n* = 1) aspirates. The dogs were a median of 8 years old (range, 2–13 years) with 20 female (2 intact) and 17 male (2 intact) dogs. Breeds consisted of 17 mixed breed dogs, 5 German Shepherds, 3 Golden Retrievers, 2 Australian Shepherds, 2 Rottweilers, and one each of the following: Belgian Tervuren Shepherd, Bernese Mountain Dog, Bichon Frise, English Springer Spaniel, Jack Russell Terrier, Shih Tzu, Vizsla, and Yorkshire Terrier. Five dogs with a moderate to marked lymphocytosis (median, 60.4 × 10^6^/mL, range, 18.1–146.8 × 10^9^/L), consisting of small to intermediate lymphocytes, were diagnosed with B-CLL. The remaining 32 dogs were diagnosed with B cell lymphoma/leukemia from lymph node or bone marrow aspirates or blood samples, with 1 concurring histologic diagnosis on a lymph node biopsy. None of the tumor cells were labeled with the anti-CD80 antibody (Figure 5, Table 6, Supplementary Figure S1, Supplementary Table S3). Of the other myeloid antigens used in this study, tumor cells were CD11c^+^ in 1/13 dogs (8%). A few dogs had aberrant CD3^+^ (7/37, 19%) or CD5^+^ (2/37, 5%) tumor cells; however, in all of these cases, tumor cells were CD21^+^ and CD22^+^, supporting a B cell neoplasm. In 3 dogs, there were discordant CD21 and CD22 reactions, with CD21^−^/CD22^+^ (n = 2) or CD21^+^/CD22^−^ (*n* = 1) cells (Supplementary Table S3). A B cell lineage was confirmed for 1 dog with a CD21^−^/CD22^+^ lymphoma/leukemia in the bone marrow on the basis of a clonal B cell population on polymerase testing for antigen receptor rearrangements (PARR) and CD20^+^/CD3^−^ cells on immunocytochemical staining of bone marrow smears (Supplementary Table S3).

**Table 6:** Positive labeling with the anti-CD80 antibody in tumor cells in dogs with hematopoietic neoplasms.

Neoplasm	Number	CD80 (*n*, %)
**B cell**	**37**	**0 (0%)**
Chronic lymphocytic leukemia	5	0 (0%)
Lymphoma/leukemia	32	0 (0%)
**T cell**	**35**	**0 (0%)**
Chronic lymphocytic leukemia: CD8^+^	2	0 (0%)
Lymphoma/leukemia	33	
CD4^+^	10	0 (0%)
CD8^+^	8*	0 (0%)
CD4^+^/CD8^+^	2	0 (0%)
CD4^−^/CD8^−^	13	0 (0%)
**Acute myeloid leukemia**	**39**	**28 (72%)**
Unclassified	1	0 (0%)
Myelomonocytic	9	6 (67%)
Myelomonocytic (cytochemistry)	1	1 (100%)
Monocytic/monoblastic	20	18 (90%)
Monocytic/monoblastic (cytochemistry)	5	1 (20%)
Megakaryoblastic	3	2 (66%)
**“Mixed lineage” leukemia**	**11**	**4 (36%)**
Myeloid antigens/CD3^+^	2	2 (100%)
Myeloid antigens/CD5^+^	1	0 (0%)
Myeloid antigens/CD22^+^	1	0 (0%)
Myeloid antigens/CD3^+^/CD5^+^/CD22^+^	1	1 (100%)
Cytochemistry/CD3^+^	1	0 (0%)
Cytochemistry/CD5^+^	3	0 (0%)
Cytochemistry/CD3^+^/CD5^+^	1	0 (0%)
Cytochemistry/CD22^+^	1	1 (100%)

^*^Includes a Golden Retriever with a granular lymphocytosis in blood and bone marrow and a concurrent lymphocytosis of CD45^−^ cells (T zone).

T cell neoplasms were identified in *35* dogs from blood (*n* = 17) or aspirates from lymph node (*n* = 11), bone marrow (*n* = 2), mediastinal or lung masses (*n* = 2), or pleural (*n* = 2) or peritoneal (*n* = 1) fluid. The dogs were a median of 6 years old (range, 8 months to 14 years) with 12 female (1 intact) and 23 male (5 intact) dogs. Breeds consisted of 9 mixed breed dogs, 6 Golden Retrievers, 3 Labrador Retrievers, 2 German Shepherds, 2 Shih Tzus, and one each of the following: Australian Shepherd, American Bulldog, Bassett Hound, Bernese Mountain Dog, Bloodhound, Boxer, Bull Mastiff, Doberman, English Bulldog, Giant Schnauzer, Mi-Ki, Pug, and Staffordshire Bull Terrier. Two dogs with a lymphocytosis of granular lymphocytes (85.3 and 89.7 × 10^9^/L) were diagnosed with CD8^+^ T-CLL. Another dog had a mild lymphocytosis of granular lymphocytes (5.5 × 10^9^/L), with an average of 39% granular lymphocytes in bone marrow. The dog (a Golden Retriever) also had a lymphocytosis (8.7 × 10^9^/L) of T cells that lacked cytoplasmic granules and were CD45^−^ on phenotyping, supporting a concurrent indolent T zone lymphocytosis. Given the bone marrow infiltrates of granular lymphocytes, this dog was placed in the CD8^+^ T cell lymphoma/leukemia category (Supplementary Table S4). Another 33 dogs were diagnosed with T lymphoma/leukemia from blood or aspirates of lymph node, bone marrow, mediastinal or lung masses, or body cavity fluid, with 2 corroborating histologic diagnosis. One dog had a clonal T cell population with PARR in a lymph node aspirate. In 2 dogs (a Bull Mastiff and a Shih Tzu), the cells were CD45^−^ and had cytologic features of an indolent T zone lymphoma (6). None of the tumor cells in dogs with T cell neoplasms were labeled with the anti-CD80 antibody (Figure 4, Table 6, Supplementary Table S4); however, the cells were CD11b^+^ (2/22, 9%) or CD11c^+^ (6/19, 32%) in low numbers of dogs. There was discordant CD3 or CD5 expression in a few cases, with CD3^−^/CD5^+^ (*n* = 4) or CD3^+^/CD5^−^ (*n* = 5) cells. Tumor cells in 2 dogs were CD3^−^/CD5^−^ but were CD8^+^/ TCRαβ^+^ on flow cytometric analysis in one dog or strongly CD3^+^ on immunocytochemical staining in the other dog (Supplementary Table S4), supporting a T cell origin.

Thirty nine dogs were diagnosed with AML based on flow cytometric expression of myeloid-associated antigens (*n* = 33) or positive cytochemical staining reactions combined with negative staining for T (CD3/CD5) or B (CD21/CD22) lymphoid-associated antigens on flow cytometric analysis (*n* = 6) (Table 6, Supplementary Table S5). The phenotyping was done on blood (*n* = 23) or aspirates of bone marrow (*n* = 10), lymph node (*n* = 5), or pleural fluid (*n* = 1). In venous blood samples, blasts constituted ≥20% of a differential count in 22 dogs (22/23, 96%). The single dog with 10% blasts in blood had ≥20% blasts on cytologic examination of a lymph node aspirate (flow cytometric analysis was not done on the lymph node). The dogs were a median of 7.5 years old (range, 2–14 years) with 11 neutered female and 28 male (5 intact) dogs. Breeds consisted of 14 Golden Retrievers, 6 mixed breed dogs, 5 Labrador Retrievers, 4 German Shepherds, 2 Pembroke Welsh Corgis, and one each of the following: Anatolian Shepherd, Bernadoodle, Bernese Mountain Dog, Bulldog, Cockapoo, Maltese, Rhodesian Ridgeback, and Soft-coated Wheaten Terrier. Tumor cells were labeled with the anti-CD80 antibody in 28 cases (72%), mostly myelomonocytic and monocytic/monoblastic variants (Figure 4, Table 6, Supplementary Figure S1, Supplementary Table S5). However, 2 acute megakaryoblastic leukemias had CD80^+^ cells (Supplementary Table S5). A higher proportion of dogs had CD80^+^ cells compared to CD4^+^ (36%, 14/39), CD11b^+^ (44%, 17/39), CD11c^+^ (46%, 16/35), CD14^+^ (38%, 15/39), or CD18^+^ (56%, 10/17) cells; however, the difference was only significant for CD80^+^ vs. CD4^+^ (*p* = 0.003) or CD14^+^ (*p* = 0.006) (Supplementary Table S5). A few individual dogs with AML expressed only one myeloid antigen (CD11b in one dog, CD11c in one dog, CD80 in 4 dogs), however most dogs expressed different combinations of more than one myeloid antigen (Supplemental Table S5).

Eleven dogs were diagnosed with “mixed lineage” leukemia based on flow cytometric expression of myeloid-associated markers (*n* = 5) or positive cytochemical staining reactions combined with positive staining for B (CD21 or CD22) or T (CD3 or 5) lymphoid-associated antigens on flow cytometric analysis (*n* = 6) (Table 6, Supplementary Table S6). The phenotyping was done on blood (*n* = 8) or aspirates of bone marrow (*n* = 1) or lymph node (*n* = 2). Venous blood in 10 dogs contained ≥20% blasts and 1 dog had ≥20% blasts in a lymph node aspirate (Supplementary Table S6). The dogs were a median of 8 years old (range, 3–10 years) with 6 female (1 intact) and 5 male (2 intact) dogs. Breeds consisted of 3 Labrador Retrievers and one each of the following: Bernese Mountain Dog, Cavalier King Charles Spaniel, Golden Retriever, Labradoodle, mixed breed, Rottweiler, Swiss Mountain Dog, and Yorkshire Terrier. The neoplastic cells were weakly CD5^+^ or CD3^+^ (*n* = 9) or CD22^+^ (*n* = 3); no cases were CD21^+^ and 1 case was weakly CD3^+^/CD5^+^/CD22^+^. In two cases with weak CD3^+^ tumor cells on flow cytometric analysis, the tumor cells were negative for CD3 on immunocytochemical staining of smears, suggesting a false positive reaction. Tumor cells were labeled with the anti-CD80 antibody in 4 cases (36%), including 1 case that lacked other myeloid-associated antigens on flow cytometric analysis (Table 6, Supplementary Table S6). CD80^+^ cells were present in similar percentages to CD4^+^ (27%, 3/11), CD11b^+^ (45%, 5/11), CD11c^+^ (33%, 3/9), CD14^+^ (27%, 3/11), and CD18 (50%, 1/2) (Supplementary Table S6).

## 4 Discussion

We found that CD80, as detected with the antibody clone in this study, is a useful flow cytometric marker for AML, particularly myelomonocytic and monocytic/monoblastic variants. This finding is in concert with the antibody labeling neutrophils, neutrophil precursors (band neutrophils to myelocytes) and monocytes in peripheral blood and bone marrow from healthy dogs. Compared to the other myeloid-associated antigens used in this study, tumor cells were CD80^+^ in a higher proportion of dogs with AML. In addition, CD80 was the only myeloid antigen expressed in some dogs categorized as AML based on cytochemical staining reactions. These leukemias would not have been diagnosed as an AML with flow cytometric analysis, since cytochemical staining is not a routinely performed phenotyping test. Similarly, in acute leukemias expressing myeloid and lymphoid-associated antigens, the presence of CD80^+^ tumor cells would support an AML. Indeed, tumor cells in 3 of 4 dogs with “mixed lineage” leukemias were positive for multiple myeloid-associated antigens, including CD80, favoring an AML with aberrant, typically weak, lymphoid antigen expression. Given that a few dogs with AML only had positive results with single myeloid antigens, our results show that antibodies against multiple myeloid antigens should be applied when immunophenotyping an acute leukemia, including CD80. Our data for CD80 in dogs contrasts with that in human patients, where CD80 is an insensitive marker of AML, being positive in <20% of cases (31, 32, 37–40). Only one study of 105 AML human patients had a higher percentage of CD80^+^ cases (33–100%) (41). However, the clone used in the studies was not always stated, making it difficult to explain discrepant results. In contrast, CD86, another member of the B7 family, is expressed on 23–90% of AML in studies of 20–110 human patients (31, 32, 37–41). CD80 can be upregulated in cultured AML cells after exposure to inflammatory cytokines (37, 42) and chemotherapeutic drugs, such as cytosine arabinoside (43). Upregulation of CD80 is speculated to promote a cytotoxic anti-tumor cell response (43).

Based on our results using the hamster anti-CD80 antibody, CD80 appears to be a specific marker for AML, whereas the other myeloid-associated antigens can be expressed in dogs with lymphoid neoplasms, as seen in this and other studies (2, 16, 33). However, continued testing of more dogs with hematopoietic neoplasia is warranted, as it is unlikely that any marker is 100% specific for AML. With a different CD80 clone (CA24.5D4), histiocytic tumors in 2 dogs (multiple cutaneous histiocytic sarcoma and dendritic cell leukemia) had positive reactions for CD80 on immunohistochemical staining (44, 45). These two reports suggest that CD80 could also be a marker of histiocytic neoplasms. In contrast to our results in dogs, in one study of 241 human patients, CD80 was expressed in 43 to 97% on B cell tumors, including diffuse large cell and marginal zone lymphoma (46), which are common subtypes of B cell tumors in dogs (47). Other studies have also shown CD80 expression on B cell tumors in humans (48–50). Unlike dogs, CD80 is expressed on human peripheral blood B cells and memory and germinal center B cells (39, 51), which would explain the positive reactions in B cell neoplasms. On the other hand, peripheral blood T cells in healthy human donors do not express CD80 (39), but positive reactions are seen in tumor cells of human patients with adult T cell leukemia/lymphoma and cutaneous lymphoma (52, 53).

Given the discrepant results in our study and reported findings in humans, it is possible that the hamster anti-CD80 antibody is cross-reacting with another member of the B7 family of molecules, such as CD74 or CD86, as found for other anti-CD80 antibodies (54). Regardless, the antibody is still detecting an antigen on neutrophils and monocytes in the blood and bone marrow of healthy dogs and on tumor cells in dogs with AML with flow cytometric analysis, which is the intended application of the antibody. Further studies, such as immunoblotting or immunoprecipitation followed by protein sequencing, would be required to determine the exact antigen detected by the hamster anti-CD80 antibody. However, these procedures are not listed in the application sheet for the antibody and the antibody may not work in denatured samples. Our results showing that the 16-10A1 clone binds to neutrophils and monocytes in healthy dog blood contrasts with previous studies. With the same clone, CD80 was not expressed in peripheral blood mononuclear cells (55), which contains monocytes (26, 27, 30), and there was no-to-weak expression in adherent monocytes in culture (27, 55). The reason for this discrepancy is unclear but may be related to technique (e.g., antibody dilution) or testing of cultured cells. We did not test other anti-CD80 antibody clones, including 1G10 and CA24.5D4. With flow cytometry, clone 1G10 labels 80% of peripheral blood monocytes and CD14-isolated monocytes after 12 days in culture, with cytokine stimulation upregulating expression intensity (26). Clone CA24.5D4 only bound to 10–20% of monocytes in canine PBMCs with flow cytometric analysis, but binding increased to more than 50% after distemper virus infection (28). These reports suggest that the 1G10, but not CA24.5D4, clone could be used to detect CD80 with flow cytometry in dogs with AML; however, this remains to be tested in future studies. Discrepant results between studies also reiterate the importance of the clone used for antigen detection and the need to provide this information in published studies. It would be worthwhile also testing an antibody against CD86 (e.g., clones CA24.3E4 or FUN-1) (26, 27, 56) in dogs with hematopoietic neoplasia as another potential flow cytometric myeloid marker. CD86 is weakly expressed on CD14-isolated canine monocytes after 7 days in culture, and higher proportions of monocytes expressed CD86 (clone CA24.3E4) vs. CD80 (clone CA24.5D4) after 1 day in culture (27), as assessed by flow cytometric analysis. Flow cytometric analysis of CD86 expression on canine hematopoietic neoplasms has not been performed to our knowledge but immunohistochemical staining with CD86 (clone CA24.3E4) yielded discrepant results (positive and negative) of histiocytic tumors in 2 dogs, both of which were also positive for CD80 (44, 45). Neither of the CA antibody clones for CD80 or CD86 are conjugated or commercially available, making it difficult to use them routinely for diagnostic purposes.

We only had low numbers of certain types of lymphoid tumors in this study, such as CLL, and continued testing of the anti-CD80 antibody for specificity in AML would be worthwhile. Several dogs with lymphoid neoplasms had ≥25% blasts in blood or bone marrow and could have been an ALL, as defined per WHO guidelines (15), vs. lymphoma. However, it is difficult to accurately distinguish between lymphoma and leukemia (being two ends of a spectrum of lymphoid neoplasia), thus we grouped the dogs with lymphoid neoplasms other than CLL as lymphoma/leukemia. It is possible that some of the dogs classified as an AML based on cytochemical staining were T-ALL. Tumor cells in T cell neoplasms can have positive staining reactions for ALP, ANBE and CAE (2, 57), reinforcing that these stains are lineage-associated and not lineage-specific. Similarly, “mixed lineage” leukemias may reflect aberrant expression of markers vs. a true mixed lineage or mixed phenotype leukemia. Aberrant marker expression, including cross-lineage antigen expression and lack of lineage-associated antigens, has been reported in AML and lymphoid neoplasms in dogs (2, 3, 16, 33). False positive reactions may also explain the weak expression of lymphoid antigens in AML cases. For instance, weak positive flow cytometric reactions for CD3 were not always corroborated by immunocytochemical staining, suggesting a false positive reaction in certain cases. We only applied the anti-human CD18 antibody (clone YFC118.3) to low numbers of cases. We had previously used another anti-canine CD18 antibody (clone CA1.4E9; Bio-Rad Cat# MCA1780A647, RRID:AB_2020973) in flow cytometric panels, but the antibody stains all blood leukocytes (29). This staining pattern contrasts with the anti-human CD18 antibody, which only stains neutrophils and monocytes in canine blood and was used in a recently proposed scheme for classification of CD34^+^ acute leukemia (16). We found the anti-human CD18 antibody was less sensitive than anti-CD80 antibody, even when these antibodies were combined with CD34 for dual labeling. However, additional comparative testing is needed. It is also possible that CD80 and other myeloid-associated antigen expression on residual normal monocytes or neutrophils contributed to the percentage of positive cells in the tumor cell gate. It is impossible to always distinguish normal leukocytes from neoplastic cells on dot plots; however, we attempted to separate out residual normal cells by comparing flow cytometric cell percentages to those in differential cell counts in modified Wright’s-stained blood or cytology smears and looking for overlap with CD14 in expected regions in FSC and SSC plots to reduce the likelihood of false positive reactions from normal cells.

## Data availability statement

The raw data supporting the conclusions of this article will be made available by the authors, without undue reservation.

## Ethics statement

The animal studies were approved by Institutional Animal Care and Use Committee. The studies were conducted in accordance with the local legislation and institutional requirements. Written informed consent was obtained from the owners for the participation of their animals in this study.

## Author contributions

TS: Conceptualization, Data curation, Formal analysis, Funding acquisition, Investigation, Methodology, Project administration, Supervision, Writing – original draft. ST: Formal analysis, Investigation, Writing – review & editing. MH: Methodology, Writing – review & editing. SZ: Data curation, Writing – review & editing.
