# Supplementary material for: Flow cytometric-based detection of CD80 is a useful diagnostic marker of acute myeloid leukemia in dogs
Source: Front Vet Sci. 2024 Aug 19;11:1405297. doi: 10.3389/fvets.2024.1405297 (PMC11366633; doi:10.3389/fvets.2024.1405297)

Supplementary Figure 1: CD80 labeling of non-tumor events in dogs with hematopoietic neoplasia. Representative flow cytometric images of venous blood in one case each of B cell lymphoma/leukemia (**A**) and acute myeloid leukemia (AML, **B**). These images are from the same dogs shown in Figure 5. Either neutrophils (**A**) or small cells (**B**) were gated in forward versus side scatter plots (first panel) and then assessed for anti-CD34-phycocerythrin (PE), CD80-allophycocyanin (APC) and major histocompatibility II-fluorescein isothiocyanate (MHCII-FITC) antibody labeling. The respective gates were separated into CD34-negative (CD34$^-$) and CD34-positive (CD34$^+$) cells using a CD34-PE versus side scatter plot (second panel). Quadrant plots of MHCII-FITC versus CD80-APC were then used to further define the CD34$^-$ cells, which were the majority of the population (third panel). Neutrophils were CD80$^+$/MHCII$^-$, as expected, in the dog with B cell lymphoma/leukemia (**A**, third panel) and the CD34- small cells were largely CD80$^-$/MHCII$^-$ (**B,** third panel), as expected for small lymphocytes. Isotype controls were used to set the quadrant regions (**A** and **B**, fourth panel).

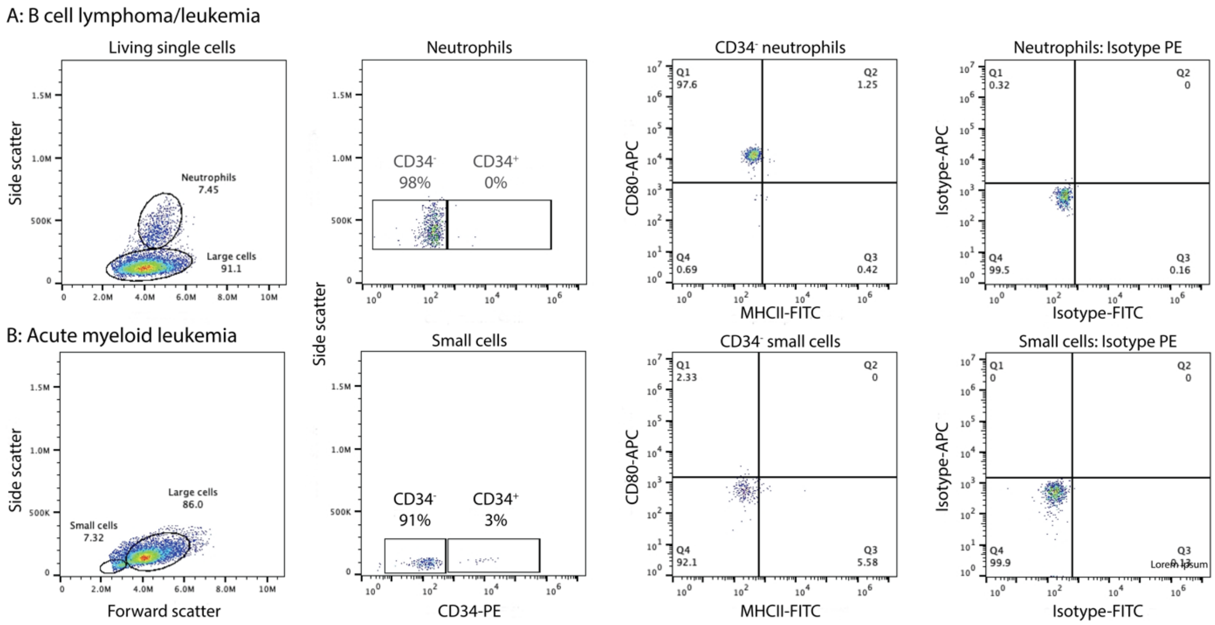

Supplement: Supplementary file 2 [file Image_1.pdf]
